# Modelling the control of bovine brucellosis in India

**DOI:** 10.1098/rsif.2022.0756

**Published:** 2023-03-08

**Authors:** H. R. Holt, M. Walker, W. Beauvais, P. Kaur, J. S. Bedi, P. Mangtani, N. S. Sharma, J. P. S. Gill, J. Godfroid, J. McGiven, J. Guitian

**Affiliations:** ^1^ Veterinary Epidemiology, Economics and Public Health Group, WOAH Collaborating Centre in Risk Analysis and Modelling, Department of Pathobiology and Population Sciences, Royal Veterinary College, University of London, Hatfield AL9 7TA, UK; ^2^ Communicable Diseases Policy Research Group, London School of Hygiene and Tropical Medicine, Keppel St, London WC1E 7HT, UK; ^3^ London Centre for Neglected Tropical Disease Research, Department of Infectious Disease Epidemiology, Imperial College London, London W2 1PG, UK; ^4^ Comparative Pathobiology Department, College of Veterinary Medicine, Purdue University, West Lafayette, IN 47906, USA; ^5^ Department of Veterinary Microbiology, Guru Angad Dev Veterinary and Animal Sciences University, Ludhiana, Punjab, India; ^6^ School of Public Health and Zoonosis, Guru Angad Dev Veterinary and Animal Sciences University, Ludhiana, Punjab, India; ^7^ Faculty of Epidemiology and Population Health, Department of Infectious Disease Epidemiology, London School of Hygiene and Tropical Medicine, London, UK; ^8^ Faculty of Biosciences, Fisheries and Economics, Department of Arctic and Marine Biology, UiT – The Arctic University of Norway, Hansine Hansens veg 18, 9019 Tromsø, Norway; ^9^ WOAH Brucellosis Reference Laboratory, FAO Collaborating Centre for Brucellosis, Department of Bacteriology, Animal & Plant Health Agency, Surrey, UK

**Keywords:** brucellosis, transmission model, zoonosis, India, dairy cattle, s19 vaccination

## Abstract

Brucellosis imposes substantial impacts on livestock production and public health worldwide. A stochastic, age-structured model incorporating herd demographics was developed describing within- and between-herd transmission of *Brucella abortus* in dairy cattle herds. The model was fitted to data from a cross-sectional study conducted in Punjab State of India and used to evaluate the effectiveness of control strategies under consideration. Based on model results, stakeholder acceptance and constraints regarding vaccine supply, vaccination of replacement calves in large farms should be prioritized. Test and removal applied at early stages of the control programme where seroprevalence is high would not constitute an effective or acceptable use of resources because significant numbers of animals would be ‘removed’ (culled or not used for breeding) based on false positive results. To achieve sustained reductions in brucellosis, policymakers must commit to maintaining vaccination in the long term, which may eventually reduce frequency of infection in the livestock reservoir to a low enough level for elimination to be a realistic objective. This work provides key strategic insights into the control of brucellosis in India, which has the largest cattle population globally, and a general modelling framework for evaluating control strategies in endemic settings.

## 1. Introduction

Brucellosis is a bacterial zoonosis imposing significant impacts on human health, livestock production and international trade of livestock products [1,2]. In 2011, the World Bank ranked brucellosis among the top 10 diseases in cattle, buffalo, sheep and goats and camelidae in terms of ‘Livestock Units Lost’ [2]. The main transmission routes for human infection are foodborne, from consumption of raw milk or unpasteurized dairy products, and direct contact with contaminated tissues (placenta, aborted foetuses, carcasses) and parturition fluids from infected livestock. Brucellosis causes acute febrile illness which, if not diagnosed and treated, can lead to chronic debilitation [2–4]. Systematic targeting of the livestock reservoir through vaccination with high-efficacy vaccines, culling of animals deemed to be infected and movement restrictions have led to elimination of the disease in several countries [5–7]. However, these strategies require significant resources and long-term commitment and are difficult to implement in areas where smallholder systems predominate. As a result, the disease remains endemic in many countries of Africa, Asia, Latin America and the Middle East where it is one of the major contributors to disability-adjusted life years (DALYs) lost as a result of foodborne illness [3,8–10].

India is the world's leading milk producer, with the world's largest bovine population, kept predominantly in smallholder systems. Bovine brucellosis is considered endemic throughout the subcontinent [8,11–14], however, high-quality epidemiological studies are lacking [15]. India is also home to approximately 18% of the world's human population and it is therefore likely that a significant proportion of the global burden of brucellosis infections in both people and cattle occurs here [16,17]. Control of brucellosis, and other cattle diseases, is particularly complex in India, where slaughter of cattle is not usually acceptable and is illegal in several states [12,15]. Brucellosis control has gained interest from Indian policymakers in recent years; a pilot programme termed the ‘*Brucella* free village’ was launched in 2016; aiming to eliminate brucellosis in milk-producing cattle and buffalo within 50 selected villages in 10 states [18]. This programme originally considered testing adult livestock in selected villages, culling seropositive buffalo and segregating cows testing seropositive and then vaccinating the remaining seronegative livestock in order to create ‘disease free’ villages [18]. In addition, a National Animal Disease Control Programme for Foot and Mouth Disease (FMD) and Brucellosis was approved by the Cabinet of the Central Government on 31 May 2019 and was in the planning stages while this study was conducted. The National Control Programme proposes vaccination as the primary control strategy; however, one of the major challenges is the procurement of sufficient vaccine doses. It is unlikely that very high coverage of the vaccine will be achieved in the short term, given current low availability, logistics of implementing large-scale vaccination campaigns, reluctance of some farmers to vaccinate as live vaccines may induce abortion in livestock and the sheer size of the bovine population to be vaccinated [8,19]. Therefore, evidence of whether vaccination at low coverage can still be effective and how best to allocate vaccine doses are particularly timely.

In order to evaluate the likely effectiveness of competing intervention strategies, we present a stochastic model for bovine brucellosis which captures both herd demographics and disease dynamics over time. The model is parametrized using data from a recent cross-sectional study conducted in 425 dairy farms in Punjab State of India, the state that produces the most cattle and buffalo milk per capita in India [13]. Previous work has demonstrated that brucellosis is widespread in dairy farms in Punjab where exposure of people in direct contact with cattle and buffaloes is common [4,20,21]. The effect of vaccinating a varying proportion of herds, and animals within herds, with (*Brucella* free village) and without (National Control Programme) implementation of test and removal at the inception of the control programme was investigated. This is the first brucellosis model that explicitly incorporates within- and between-herd transmission in a setting where the control programme does not include movement restrictions, which are difficult to implement in most endemic settings.

## 2. Material and methods

### 2.1. Disease transmission model

**Figure 1.**
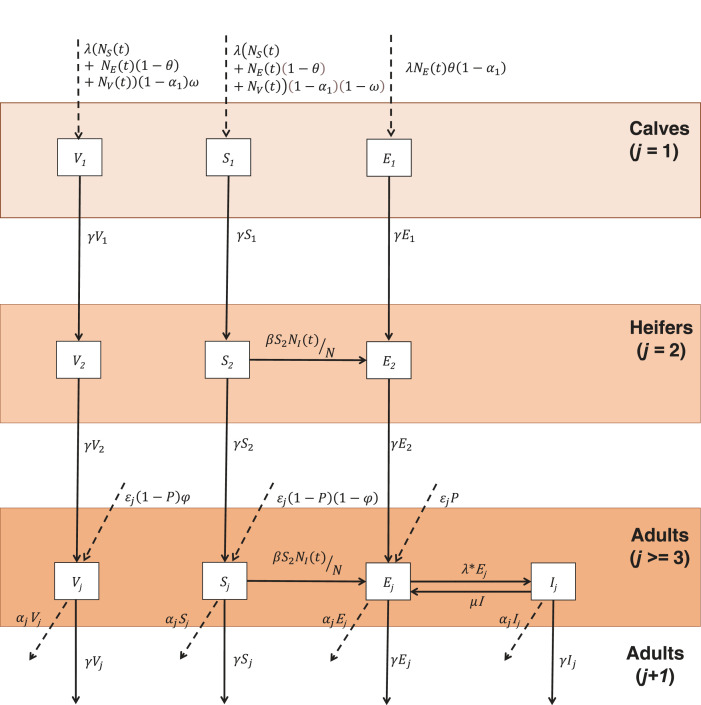
Model schematic showing the transition between different compartments, where $N_{S}(t)=\sum_{\;j=3}^{\;j=11}S_{j}(t)$, $N_{E}(t)=\sum_{\;j=3}^{\;j=11}E_{j}(t)$ and $N_{V}(t)=\sum_{\;j=3}^{\;j=11}V_{j}(t)$ denote, respectively, the total number of susceptible, exposed and vaccinated adults in the herd; *λ*, *θ*, *α*_1_ and *ω* denote, respectively, the calving rate, probability of vertical transmission, removal rate of newborns and probability a calf is vaccinated and becomes immune; *γ* is the transition rate between age groups (1 year); *β* is the effective contact rate; ε*_j_*, *P*, φ, *μ* and *α_j_* are the number of new purchases (ε*_j_*, *j* = 4), the probability that a purchased animal is infected (*P*) and vaccinated (φ), the rate of loss of infectiousness (*μ*) and the rate of removal of adults (*α_j_*).

A dynamic age-structured stochastic compartmental model incorporating dairy herd demographics was written in R [22] to capture transmission of *B. abortus* within and between dairy herds. As the model is used to capture dynamics in dairy herds typical of Punjab State of India, large ruminants (cows/buffalo) are the only species considered as very few farms or households in this area keep small ruminants (2%) [13]. In addition, *B. abortus* is the only species to be isolated from large ruminants here [13]. Cows and buffalo are assumed to mix homogeneously within the herd as management practices in Punjab are very similar and farms often keep a mixture of both species. Transition between compartments follows a Poisson process using the event-driven Gillespie stochastic simulation algorithm (SSA) implemented using the R package GillespieSSA [23]. The model tracks the numbers of livestock in each state; susceptible (*S*_i_), infected (*E*_i_), vaccinated (*V*_i_) (all age groups) and infectious (*I*_i_; adults only), as well as vaccine doses and the number of animals ‘removed’ from the dairy herds when implementing test and removal strategies (figure 1 and electronic supplementary material).

### 2.2. Model events

Replacement animals are modelled as being sourced either from new births on the farm, with *λ* denoting the annual calving rate, or from the purchase of new animals of age group *j* = 4 (ε*_j_*). Male calves, new-borns sold off the farm or calves that die in their first year of life cannot become infectious while on the farm and therefore do not contribute to transmission; these events are incorporated in a single rate parameter, *α*_1_*.* In other age groups, livestock die, are sold or are no longer reproductively active and these events are captured by parameter, *α_j_*. Of those calves remaining in the farm, those born to infected cows can be infected (exposed), *E*_1_, via vertical transmission from mother to calf, with probability *θ.* When calfhood vaccination is implemented, a proportion of calves enter the *V*_1_ compartment with probability *ω*. The remaining calves enter the *S*_1_ compartment.

Susceptible cattle become infected following an effective contact with infectious cattle, governed by (transmission rate) parameter *β*. Infected cattle become infectious when they abort or give birth (rate *λ*). Following a period of infectiousness, capturing shedding of the infectious cattle and the survival of *B. abortus* organisms in the environment, cattle return to the infected (exposed) compartment at rate *μ* until they give birth again. Transmission of *Brucella* spp. is via proximity to calving livestock, contaminated environments, *in utero* or during service/artificial insemination. Therefore, as herds in Punjab are primarily sedentary and effective contacts are unlikely to occur between livestock from different farms [13], it is assumed that between-herd transmission only occurs through purchase of infected cattle. This is modelled as a function of the number of annual purchases (ε*_j_*) and the probability that a replacement animal is infected, as given by the animal-level prevalence, *P*.

### 2.3. Model parametrization and assumptions

The model is parametrized using data from a sero-survey of 425 dairy herds in rural Ludhiana district of Punjab conducted between 2015 and 2017; 139 herds had at least one positive animal (median within-herd prevalence 33.3%) [19]. These data are supplemented with information collected from a survey of 409 household herds [21] and published literature. Infection in a farm is seeded from the purchase of infected animals. Two zero-inflated negative binomial distributions for purchase numbers were fitted using maximum-likelihood estimation implemented with the R package fitdistrplus [24] to data on the number of purchases per farm per year; one for herds with less than nine adult females and one for herds with nine or more females. The effective contact (transmission) rate within a farm, *β*, was fitted using approximate Bayesian computation (ABC) techniques.

The model parameters are listed in table 1. As there was high uncertainty associated with the probability a calf born to an infected animal is infected *θ* and this could potentially influence which control strategies are considered most effective this parameter was varied as part of a scenario analysis. See table 1, electronic supplementary material and Holt *et al.* [13] for further details.

**Table 1.** Model parameter definitions and values.

parameter	definition	value	source
*λ*	calving rate (and rate that exposed animals become infectious)	0.605 per year	[19]
*γ*	transition rate between age groups	1 per year	
ε*_j_*	rate of purchasing new animals of age group *j*	sampled for *j* = 4; 0 otherwise	estimated from observed data [19]
*α*_1_	rate of removal of newborn calves (composite parameter capturing male calves, death and sale of calves)	0.685 per year	[19]
*α_j_*	rate of removal of adults (composite parameter capturing death, sale and end of reproductive activity of adults)	12/72 for *j* = 3 … 10 0.835 for *j* = 11	estimated from age distribution [19]
*β*	cattle-to-cattle effective contact (transmission) rate	5.279	this work
*μ*	rate of loss of infectiousness (composite rarameter capturing period bacteria are shed plus their survival in the environment)	12/4 per year	[25,26]
*θ*	probability calf born to infected animal is infected	0.05 (0.2 in scenario analysis)	[27–29]
*ω*	calves which are immune (proportion vaccinated ∗ VE)	varied value	proportion vaccinated = 0.25; 0.5; 0.75 or 1.0 depending on scenario
φ	probability that a purchased animal is vaccinated	varied value	initially 0, updated annually when simulating control strategies
*P*	probability that a purchased animal is infected	initially 0.151. Then varied as control strategies are implemented.	[19]
VE	vaccine efficacy	0.8	[30]
Se	test sensitivity Rose Bengal test (RBT)	0.9	[31]
Sp	test specificity RBT	0.9	
	proportion of animals that are buffalo (versus cows)	0.45	[19]

### 2.4. Fitting cattle-to-cattle effective contact (transmission rate)

**Figure 2.**
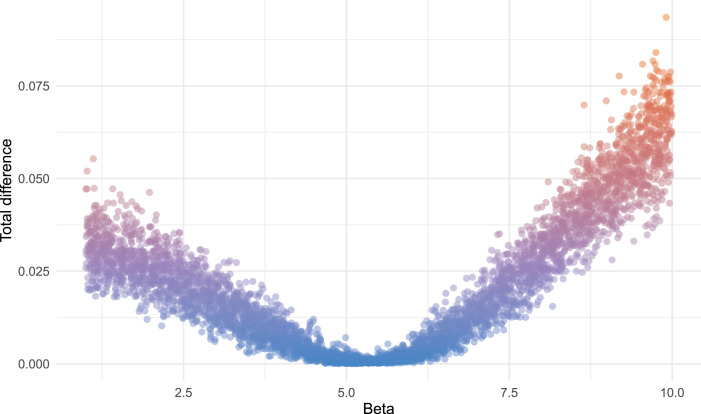
Scatterplot showing the sum of squared difference between the mean and standard deviation of the observed and simulated herd prevalence data for each value of the cattle-to-cattle effective contact (transmission) rate parameter *β* (beta).

The rate that susceptible cattle become infected is driven by the effective contact rate, *β*. This was estimated by fitting the model to the observed within-herd seroprevalence data presented in Holt *et al*. [13] using an approximate Bayesian computation (ABC) algorithm. The algorithm samples a value of *β* from a prior distribution and then produces a stochastic simulation of the prevalence of infection for each of the 425 observed herds (after a burn-in period of 75 years to ensure endemic stability). An uninformative uniform prior bounded between 1 and 10 was used for *β* and 5000 values were sampled from this distribution. Initial herd sizes for each simulated herd were set to match the corresponding observed herd size and all animals within herds were initially assumed to be uninfected (i.e. infection introduced from the purchase of infected animals). Infections were seeded through the purchase of new animals. The sum of the squared difference between the observed and simulated mean and standard deviation of the within-herd prevalence values was used as the distance measure to minimize (figure 2), with differences less than 0.005 retained for approximation of the posterior. The median value of *β* (=5.279) was selected as a fixed parameter value for simulations of different control strategies for computational feasibility.

### 2.5. Simulation of control scenarios

Simulations of the impact of different control scenarios were based on vaccinating farms within villages, with ‘village’ being the unit considered for control by the *Brucella* free village and National Control programmes. The mean population of a village and household size in the study area is approximately 1500 and 5.2 people, respectively [32] and with 61.0% of households keeping cattle [21]. Therefore, it was assumed an average village would contain 180 herds. Median herd size in the study area was four (2.5th–97.5th percentile; 1–16) with a maximum of 24 animals [19]. Therefore, initial herd sizes for 180 dairy herds per village were sampled from zero-truncated negative binomial distribution fitted to the herd size data, and the model was run for 75 years to stabilize.

Control scenarios were selected based on interventions proposed by the *Brucella* free village programme (test and removal at the inception of the control programme and subsequent vaccination) and the National Control Programme (vaccination only). Hence, the model was used to investigate the impact of different combinations of the following:

#### 2.5.1. Vaccination strategies

(i) vaccinating livestock within different subsets of herds within villages. A: all herds; B: 50% of herds (randomly selected); C: large herds only (9+ animals)
(ii) vaccinating different proportions of livestock (all age groups) within vaccinated herds at the inception of the control programme (year 1) (0, 0.5 or 1)
(iii) vaccinating different proportions of calves within vaccinated herds annually (0.25, 0.5, 0.75 or 1)

Within vaccinated herds (i), it is assumed that vaccination of ‘all age groups’ (ii) occurs at a single time point once ‘endemic stability’ has been reached. A proportion (*VE* ∗ proportion vaccinated) of *S*_j_ animals move into the *V*_j_ compartments at the inception of the control programme and remain there until they leave the herd. Calfhood vaccination (iii) strategies are simulated by varying parameter *ω* in the within-herd model.

#### 2.5.2. ‘Test and removal’

Scenarios combining calfhood vaccination with testing of all animals (cows and buffalo of all age groups using the Rose Bengal test) and immediate removal of test positives were also simulated. Test and removal is assumed to occur at a single point in time, at the inception of the control programme (as proposed in the *Brucella* free village programme). There is no differentiate infected from vaccinated animals (DIVA) test available for brucellosis, therefore this strategy was not combined with vaccination at the inception of the programme. The number of true test positive animals is sampled from a Bernoulli distribution with the number of trials equal to *E*_j_+ *I*_j_ and the probability of testing positive given by the test sensitivity, Se. False positive animals are also sampled from a Bernoulli distribution with number of trials equal to *S*_j_ and the probability of giving a false positive result equal to 1 − Sp*.* Scenario B was not simulated with test and removal as it was not considered an effective use of resources compared with Scenario C (see Results).

Targets for control were set using prevalence (percentage of infected animals in a village), as this is measurable via surveillance. Therefore, we define ‘control’ as animal-level prevalence below 1%, as indicative of a nominally low level of infection whereby Punjab could consider moving towards elimination. The ‘probability of control’ is the percentage of simulations with prevalence less than 1%. Predicted (cumulative) incidence, the percentage of animals infected with *B. abortus* per year, is also presented.

## 3. Results

### 3.1. Scenarios without ‘test and removal’

**Figure 3.**
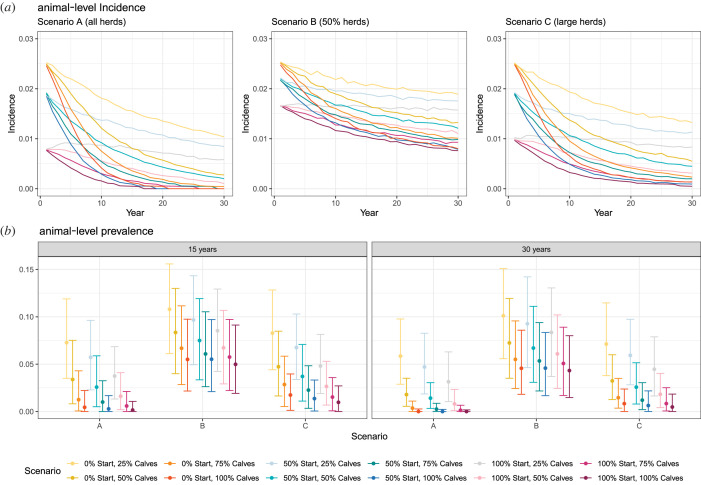
Incidence (*a*) and prevalence (*b*) of bovine brucellosis during 30 years of simulated control programmes. Different intervention strategies are indicated by different colours on the legend, where ‘start’ indicates the percentage of animals within herds (all ages) that are vaccinated in the first year and ‘calves’ indicates the percentage of calves that are vaccinated annually thereafter. All results are based on 1000 iterations (repeat stochastic simulations) per village. Scenario A, all herds selected for vaccination; Scenario B, 50% of herds randomly selected for vaccination; Scenario C, large herds (9 + animals) selected for vaccination.

**Figure 4.**
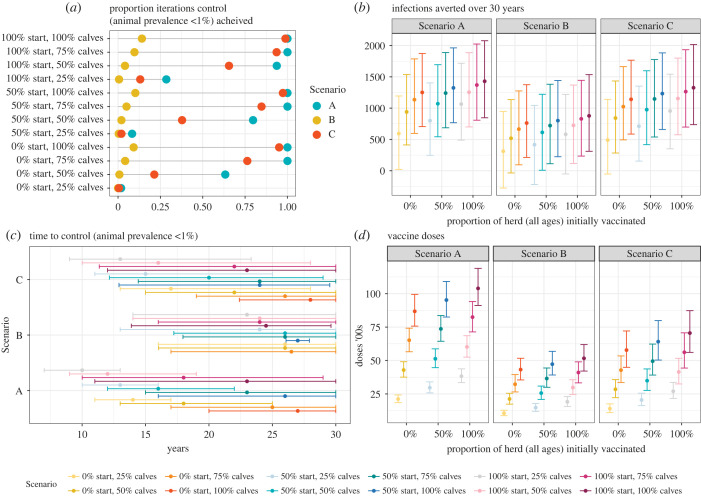
Summary of the effectiveness of different vaccination strategies for controlling brucellosis. Probability ‘control’ (defined as animal-level prevalence below 1%) is achieved (*a*), number of infections averted per village (compared with baseline scenario of no control) (*b*), time to achieve control (*c*) and number of vaccine doses (*d*) after simulating each strategy for 30 years, based on 1000 iterations. ‘start’ refers to the percentage of the herd (all ages) vaccinated in year 1 of the control programme and ‘calves’ refers to the percentage of calves vaccinated annually. Median and 95% credible intervals are presented in (*a*), (*b*) and (*d*). Scenario A, all herds are selected for vaccination; Scenario B, 50% of herds randomly selected for vaccination; Scenario C, large herds (9 + animals) selected for vaccination.

The most aggressive Scenario A strategies—which involved vaccinating all herds within a village and up to 100% of replacement calves—could reduce the incidence and median prevalence of *B. abortus* to below 1% within 30 years, providing at least 75% of calves were vaccinated (figures 3*a*,*b*, 4*a*). Control (less than 1% prevalence within 30 years) could not be achieved in any of the Scenario B strategies where 50% of herds were chosen at random to be enrolled in the vaccination programme, irrespective of coverage (less than 20% probability of control; figures 3*b*, 4*a*). Vaccination of animals in herds with nine or more animals (Scenario C), where 100% or 75% of calves were vaccinated annually, resulted in control with at least 75% probability (figures 3*b*, 4*a*). Scenarios A and C averted the most bovine infections, with a median of over 1000 infections averted per village during the 30-year control period when 75% or 100% of replacement calves were vaccinated (figure 4*b*). Employing Scenario C, as opposed to Scenario A, was estimated to delay control by a median of 5 years (figure 4*c*). However, Scenario C reduces the vaccine doses by around a third and saves additional resources, as only 35% of farms need to be vaccinated (as opposed to 100%; figure 4*d*).

### 3.2. Scenarios with ‘test and removal’

**Figure 5.**
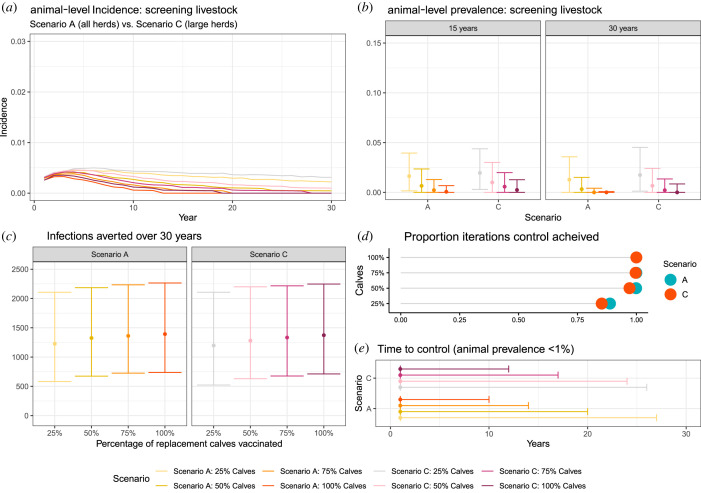
Summary of the effectiveness of combining ‘test and removal’ at the inception of the control programme combined with different calfhood vaccination strategies for controlling brucellosis. Simulated cumulative incidence over time in intervention strategies where seropositive animals are removed in year 1 (*a*), prevalence (proportion of infected bovines) after employing each strategy for 15 and 30 years (*b*), number of infections averted per village (compared with baseline scenario of no control) (*c*), probability ‘control’ (defined as animal-level prevalence below 1%) is achieved (*d*) and time to achieve control in a village (*e*). In all scenarios, test and removal is performed in year 1, and model is run over 30 years for 1000 iterations/villages ‘calves’ refers to the % of calves vaccinated. Median and 95% credible interval are presented in (*b*), (*c*) and (*e*), where Scenario A = all herds are selected for vaccination and C = large herds (9 + animals) selected for vaccination.

The impact of combining vaccination of calves within all herds (Scenario A) and large herds (Scenario C) with testing and removal of positive adults at the inception of the control programme was also investigated. These simulations resulted in control (animal-level prevalence within a village less than 1%) with more than 85% probability (figure 5*d*), within a median of 1 year (figure 5*e*). However, under the conservative assumption of a test sensitivity and specificity of 90%, these scenarios did not remove all infected livestock in year 1 (figure 5*a*) and would result in the removal of large numbers of uninfected animals (false positives). In these scenarios, a median of 102 (95% credible interval, CI: 82–132) uninfected and 141 (95% CI: 91–207) infected animals were removed per village (approx. 20% of the village population; 7 uninfected animals per 10 infected removed). Over the 30-year period, the number of new infections averted per village ranged from 1197 in Scenario C, where 25% of replacement calves were vaccinated, to 1393 in Scenario A, where 100% of calves were vaccinated. Compared with the equivalent vaccination-only scenarios, the addition of test and removal resulted in substantial reductions in new cases in scenarios with low vaccination coverage (e.g. 807 fewer infections in Scenario C with 25% of replacements vaccinated). However, in scenarios with higher coverage, similar reductions in new infections were achieved (142 fewer infections in Scenario A with 100% of replacements vaccinated) (figure 5).

## 4. Discussion

Despite successful elimination of bovine brucellosis in some countries, its control in many settings, including India, remains elusive [8]. Lack of management options for positive animals is often perceived as one of the main barriers to controlling brucellosis in India [19,33]. However, this study demonstrates that control (defined here as animal-level prevalence below 1%) of *Brucella* infections in bovines in endemic areas can be achieved through long-term vaccination, without the need to vaccinate all animals or implement a ‘test and removal’ policy. Although control, as opposed to elimination, will not result in the attainment of official brucellosis free (OBF) status, a marked decrease in the incidence of infection can be expected to result in comparable reductions in financial losses to farmers and the incidence of human infection [34–41]. Sustainability, due to limited resources and political will, has constrained brucellosis vaccination campaigns in many contexts [5,42]. Due to the chronic nature of the disease and the long lifespan of cattle in India, if vaccination campaigns are to be an effective use of resources the commitment must be long term, the goal (e.g. sustained reduction/control versus elimination) should be achievable and the control strategy realistic. The results suggest that calfhood vaccination is likely to be successful at lowering the animal prevalence of the infection below 1%. However, even with the most aggressive vaccination strategy (vaccination of 100% of animals at the start of the control programme and 100% of calves annually) the median time estimated to achieve this was 10 years, with 95% of estimates falling between 8 and 14 years. The National Control Programme, which envisages 100% vaccination of female cattle and buffalo calves once in their lifetime, has initially been budgeted for 5 years [18]. However, our results indicate that it would take a median of 14 years to achieve control when only calves are vaccinated. Furthermore, vaccination is unlikely to result in elimination, therefore calfhood vaccination would need to be implemented indefinitely to prevent re-emergence of brucellosis [5].

Strategies applied to complex settings require adaptation and should consider the diversity of the livestock systems, infrastructure and available resources. A lesson from previous brucellosis control programmes in other countries is that test and slaughter should not be introduced too early, without the resources for compensation to farmers, or where the baseline prevalence is so high that a large proportion of the livestock population requires culling [3,5,43]. For example, in the European Union, legislation related to control and eradication programmes of bovine brucellosis started in 1962, and only seven out of 15 Member States were recognized as OBF in 1999, 37 years after inception [44]. In India, the *Brucella* free village programme initially included a component on test and removal of infected livestock. If this was implemented, elimination would be accelerated with animal prevalence projected to go below 1% within the first year. However, given that 12.9% (95% CI: 9.2–17.6) of animals in the study area were estimated to be seropositive at the start of the control programme, huge resources would be required to identify these animals and remove them from the population, especially since the *Brucella* free village programme proposes to maintain infected cows in an area separate from the village [18]. In addition, replacement livestock would need to be sourced externally which may result in infected livestock being reintroduced in the village [45]. Alternatively, if sustained reductions in prevalence were achieved through targeted vaccination, then reallocation of resources towards elimination activities in certain farms, villages, states or at a national level may become feasible.

Stakeholder consultation revealed that vaccine availability is a limiting factor and one of the major activities of the National Control Programme will be vaccine procurement [19]. The situation is likely to become even more challenging as Indian Immunologicals—who introduced the brucellosis vaccine ‘Bruvax’ to the Indian market—are the primary manufacturer a SARS-CoV-2 vaccine [19,46,47]. The SARS-CoV-2 pandemic also disrupted supply chains for reagents and consumables, having caused unpredictable shutdowns of manufacturing and movement restrictions [48,49]. Policymakers should ensure that brucellosis vaccine programmes are resilient to such shocks prior to implementation. In addition to the difficulties and costs associated with the manufacture and procurement of the vaccine, deployment faces logistical issues. The most successful strategy for brucellosis control may not be the most efficient use of resources [50]; policymakers can use the information presented here to assess which vaccination scenarios will be optimal based on available doses and resources. For example, vaccinating 100% of calves under Scenario C (targeting large herds only) and Scenario A (all herds) with no test and removal and no initial vaccination was estimated to save similar numbers of infections per village (median 1326 versus 1430 over 30 years) and resulted in the control of brucellosis (animal-level prevalence <1%) in 100% of simulations. However, the former required around 30% fewer vaccine doses and only animals within about one-third of herds to be vaccinated. As well as increased herd sizes, this finding is driven by the higher rate of purchasing of new livestock in these farms, therefore screening of animals prior to sale may also present a potential future control option [19]. Stakeholders envisioned calfhood vaccination being more acceptable to farmers and veterinary offices due to the negative effects of the vaccine that can occur in adult livestock and the potential to reduce needlestick or other injuries [19]. They also reported that larger commercialized farms would be easier to target due to their heightened awareness of the disease and willingness to invest in herd health.

The aim of this study was to predict the *relative impact* of different control scenarios; hence the parameters were mostly fixed to maximize comparability. However, there is uncertainty associated with these parameters, particularly the probability a calf born to an infected cow is infected (*θ*). This was explored in sensitivity analysis using a worst case (20%) estimate which did not alter the relative effectiveness of the different scenarios [19] (electronic supplementary material). The same calving rates were used for both susceptible and infected livestock, as there is little high-quality data comparing abortion rates with *Brucella* infection in highly endemic settings. Although this may result in an overestimate of infected calves in the herd, this was considered negligible, as the probability of horizontal transmission was only 5% (20% for scenario analysis), and most infected animals do not abort more than once in their lifetime [12]. A conservative estimate for test performance (90% sensitivity and specificity, Rose Bengal test) was used; however, it is likely that accuracy, particularly specificity, is higher, which may have led to an overestimate of the number of susceptible animals giving false positive results [19]. However, even with perfect test specificity, large numbers of true positive animals (median 141 (95% CI: 91–207) per village) would still need to be culled or segregated and it would be difficult to source sufficient disease-free animals to replace those removed. Test and removal strategies are most useful in low-prevalence settings [5]. As the results already suggested that test and removal was not an effective use of resources, using these values for test performance, it was not necessary to simulate further scenarios for diagnostic accuracy.

A vaccine efficacy of 80% was used throughout; however, a recent systematic review and meta-analysis indicate this may be optimistic [51]. The S19 vaccine at a dose of 10^9^ colony-forming units (CFUs) has been estimated to be 75% (95% CI: 48–88%) and 72% (95% CI: 30.9–84%) efficacious against abortion and infection, respectively [51]. It was also found that a dose of 10^9^ CFU had the highest vaccine efficacy, which is 50–80 times lower than the dose currently recommended by the World Organisation for Animal Health (OIE) for subcutaneous administration. If the lower dose was shown to be efficacious in this setting, it could also help address the issue of lack of vaccine doses. Control was still achieved in Scenarios A and C when 60% (0.8 efficacy × 0.75 coverage) of calves enter the vaccinated compartment (greater than 75% probability for all scenarios). Therefore, a lower vaccination efficacy than the one used for this analysis could still be effective providing high enough coverage is achieved.

The model used here includes several components omitted in previous simulations of control strategies, allowing for stochastic extinction within a herd and contact rates between livestock to vary by explicitly modelling within- and between-herd transmission. A novel method of the seeding of infected livestock through trade is used, and infected animals become infectious following a calving/abortion event, as opposed to at a fixed rate. This achieves more realistic simulations, making the outputs more applicable to the issue of sustainable brucellosis control in complex settings. As with all compartmental models, waiting times between contiguous compartments are assumed to be exponentially distributed. Although this means some events can occur very quickly and others very slowly, on average the waiting time is given by the reciprocal of the associated rate parameter (e.g. the average time between calving is around 18 months). Other important assumptions include that the probability of a purchased animal being infected is equivalent to the current farm-level prevalence in the region (i.e. no infections introduced from outside of the region) and that vaccine immunity is lifelong. The former is justified based on a survey of 413 farms which found that no bovines were purchased from outside Punjab and only 10% were purchased from another district. As the National Control Programme for Brucellosis is being carried out at a large scale, we implicitly assumed that replacement animals will be sourced locally or from an area also undergoing control activities [13]. Lifelong vaccine-induced immunity was informed by past studies in the US and UK which suggest that S19-induced immunity lasts for at least five pregnancies, the normal milking length of cow [52]. Finally, cattle and buffalo are assumed to have similar management and contribution to transmission, as estimates of reproductive parameters and the odds of seropositivity did not differ between species in this setting [13], and hence there is no evidence to support their separation in the model. There are limited data on differences in susceptibility and transmissibility of *B. abortus* between the species, and differences have mostly been attributed to management or the *B. abortus* strain [53,54].

Potential further applications of the model include simulation of additional control strategies and further testing model assumptions, including diagnostic test performance and vaccine efficacy. The model can be adapted to simulate brucellosis control in other settings where both within- and between-herd transmission is important and where within-herd seroprevalence data are available. In settings where *B. melitensis* is circulating in small ruminants and cattle, then small ruminants could be considered in such modelling exercises as they are considered reservoirs for this species [55]. The National Brucellosis Control Programme presents an opportunity for data to be gathered on the implementation and the effectiveness of brucellosis control programmes which could be used to refine modelling assumptions. In addition, future data from the control programme in Punjab could be used for model validation, assessing the model's predictive performance.

In conclusion, taking Punjab State of India as a case study, this work demonstrates the application of a stochastic mathematical transmission model to evaluate the effectiveness of options for controlling bovine brucellosis, a livestock zoonosis that exerts a heavy global health and economic burden [2,56]. The results suggest that control can be achieved through targeted vaccination of larger herds without the need to achieve perfect coverage. Targeting animals within large herds and replacement calves to receive vaccination will probably be a more efficient use of resources than blanket vaccination, which is not feasible while vaccine doses are limited. However, for control to be sustained, policymakers and funders must commit to long-term vaccination campaigns beyond currently proposed timeframes of current programmes. Indeed, in the absence of additional control measures, it would be necessary to pursue vaccination indefinitely, as the model suggests elimination (prevalence close to 0) is unlikely to be achieved using vaccination-only strategies. It is imperative that stakeholders are aware of this before vaccination campaigns are implemented. Test and removal of livestock, although being efficient in reducing animal prevalence, is unlikely to be an acceptable strategy—particularly at the outset of intervention—for livestock owners in Punjab. However, once prevalence reaches a low enough level with vaccination, stakeholders could consider test and slaughter to move toward elimination.

## Data Availability

The model is available in an open source repository: https://bitbucket.org/hannah_holt/brucellosis/src/master/. The data are provided in electronic supplementary material [57].
